# Bacteremia due to *Staphylococcus cohnii* ssp. *urealyticus* caused by infected pressure ulcer: case report and review of the literature

**DOI:** 10.1590/S1516-31802013000100010

**Published:** 2013-02-01

**Authors:** Jonathan Soldera, Wagner Luis Nedel, Paulo Ricardo Cerveira Cardoso, Pedro Alves d’Azevedo

**Affiliations:** I MD. Resident, Department of Internal Medicine Service, Hospital Nossa Senhora da Conceição (HNSC) and Gastroenterology Service, Santa Casa de Misericórdia de Porto Alegre, Porto Alegre, Rio Grande do Sul, Brazil.; II MD. Resident, Department of Internal Medicine, Hospital Nossa Senhora da Conceição, and Intensive Care Service, Hospital de Clínicas de Porto Alegre, Rio Grande do Sul, Brazil.; III MD. Physician, Intensive Care Service, Hospital de Clínicas de Porto Alegre, and Preceptor, Internal Medicine Service, Hospital Nossa Senhora da Conceição, Porto Alegre, Rio Grande do Sul, Brazil.; IV PhD. Associated Professor, Universidade Federal de Ciências da Saúde de Porto Alegre (UFCSPA), Porto Alegre, Rio Grande do Sul, Brazil.

**Keywords:** Staphylococcal infections, Pressure ulcer, Bacteremia, Dementia, Community-acquired infections, Infecções estafilocócicas, Úlcera por pressão, Bacteriemia, Demência, Infecções comunitárias adquiridas

## Abstract

**CONTEXT:**

Coagulase-negative staphylococci are common colonizers of the human skin and have become increasingly recognized as agents of clinically significant nosocomial infections.

**CASE REPORT:**

The case of a 79-year-old male patient with multi-infarct dementia who presented systemic inflammatory response syndrome is reported. This was attributed to bacteremia due to *Staphylococcus cohnii* ssp. *urealyticus*, which was grown on blood cultures originating from an infected pressure ulcer. The few cases of *Staphylococcus cohnii* infection reported in the literature consist of bacteremia relating to catheters, surgical prostheses, acute cholecystitis, brain abscess, endocarditis, pneumonia, urinary tract infection and septic arthritis, generally presenting a multiresistant profile, with nearly 90% resistance to methicillin.

**CONCLUSIONS:**

The reported case is, to our knowledge, the first case of true bacteremia due to *Staphylococcus cohnii* subsp. *urealyticus* caused by an infected pressure ulcer. It shows that this species may be underdiagnosed and should be considered in the differential diagnosis for community-acquired skin infections.

## RESUMO

**CONTEXTO:**

*Staphylococcus* coagulase-negativos, colonizadores frequentes da pele humana, têm sido reconhecidos como agentes de infecções nosocomiais.

**RELATO DE CASO:**

Relata-se o caso de um paciente de 79 anos com demência vascular que apresentou síndrome da resposta inflamatória sistêmica atribuída a bacteremia por *Staphylococcus cohnni* ssp. *urealyticus*, que cresceu em hemoculturas, secundária a uma úlcera de pressão infectada. Os poucos casos de infecção por *Staphylococcus cohnii* relatados na literatura descrevem bacteremia associada a cateter, próteses cirúrgicas, colecistite aguda, abscesso cerebral, endocardite, pneumonia, infecção do trato urinário e artrite séptica, geralmente apresentando um perfil de multirresistência, com aproximadamente 90% de resistência à meticilina.

**CONCLUSÕES:**

O caso relatado é, ao nosso conhecimento, o primeiro de bacteremia verdadeira por *Staphylococcus cohnii* subsp. *urealyticus* causada por uma úlcera por pressão, mostrando que esta espécie pode estar subdiagnosticada e deveria ser considerada no diagnóstico diferencial das infecções cutâneas adquiridas na comunidade.

## INTRODUCTION

Coagulase-negative staphylococci (CoNS) are common colonizers of the human skin and are the most frequent constituent of the normal flora at this site. Although once considered to be non-virulent and probably contaminant microbes, these organisms have become increasingly recognized as agents of clinically significant nosocomial infections. Hence, it has become more important to know about the epidemiology and pathology of the species individually.

A review of the literature was conducted through an online search of keywords and the word *Staphylococcus cohnii* in the PubMed, SciELO and Lilacs databases. Relevant papers were analyzed and included in this paper, and these were mostly other case reports (Table 1).

**Table 1.** Database search results for *Staphylococcus cohnii* ssp. *urealyticus* ^*^

Database	Search terms	Results
PubMed	Bacteremia AND ((Staphylococcus AND urealyticus) OR (Staphylococcus AND Cohnii))	1 case report 12 original articles
Lilacs	Staphylococcus AND cohnii AND urealyticus	None
	Staphylococcus E cohnii E urealyticus	1 case report 2 original articles
	Staphylococcus Y cohnii Y urealyticus	1 original article
SciELO	Staphylococcus AND cohnii AND urealyticus	1 case report 1 original article
Embase	Staphylococcus AND cohnii AND urealyticus	None

^*^Search was performed on August 15, 2011.

## CASE REPORT

A 79-year-old male patient with a previous diagnosis of multi-infarct dementia and hypertension was admitted to the emergency department of Hospital Nossa Senhora da Conceição (HNSC) after an episode of malaise, shivering, fever, disorientation and hypoxemia, compatible with an episode of bacteremia, which was witnessed by a primary healthcare physician.

The patient was admitted to the emergency department with acute worsening of his baseline state of disorientation, which was fluctuating between drowsiness and agitation (diagnosed as delirium by the medical staff), tachycardia, tachypnea and mild fever, associated with a grade III infected sacral pressure ulcer (i.e. down to the superficial fascia) and pressure ulcers above both femoral trochanters (without signs of infection). Hematological and biochemical laboratory tests showed pH 7.42, PaCO_2_ 39.2 mmHg, lactate 0.9 mmol/l, leukocyte count of 15,040 with 83.3% neutrophils, and normal renal and hepatic function tests. A diagnosis of systemic inflammatory response syndrome was made, with bacteremia as the most likely cause. An active search for the focus of the probable infection found sterile urine, no sign of recent lung infection and two positive samples from peripheral blood cultures for multisensitive *Staphylococcus cohnii* ssp. *urealyticus* (which was sensitive to gentamicin, tetracycline, sulfamethoxazole/trimethoprim, cefalotin, ciprofloxacin, erythromycin, oxacillin, clindamycin and vancomycin). There was no bone exposure in the infected ulcer scar, and a normal roentgenogram ruled out local osteomyelitis. A diagnosis of true bacteremia due to *Staphylococcus cohnii* ssp. *urealyticus* caused by an infected pressure ulcer was made.

The patient was discharged from the hospital after a 10-day course of treatment: four days of empirical vancomycin and six days of antibiogram-oriented oxacillin, with regression of fever for more than 72 hours, normal leukocyte count, C-reactive protein and an improvement in clinical state, in accordance with the statement from the local Infection Control Committee for coagulase-negative staphylococci bacteremia.

The patient was admitted to hospital again, two and six months later, with pressure ulcer infections due to Gram-negative bacilli. These were treated with piperacillin/tazobactam and meropenem, respectively, with no Gram-positive cocci identified. At the last hospital admission, because of a deeper ulcer scar, the patient underwent a bone biopsy that ruled out osteomyelitis.

## DISCUSSION

Data collected in medical intensive care units through the National Nosocomial Infections Surveillance (NNIS) system between 1992 and 1997 revealed that CoNS accounted for 36% of all bloodstream isolates, making these organisms the most common cause of nosocomial bloodstream infections.^1^ A second database of nosocomial bloodstream infections in United States hospitals from 1995 to 2002 also identified CoNS as the most common cause, responsible for 31% of cases.^2^ Despite the high frequency of bloodstream infections (BSI), true bacteremia due to CoNS is an unusual event. Only 4 to 12% of patients with BSI are estimated to have this condition.^3^ Whereas contamination of blood cultures leads to additional laboratory tests, unnecessary antibiotic use and increased hospital stay, failure to recognize true bacteremia can lead to increased morbidity and mortality.

It has been estimated that more than 80% of CoNS are resistant to methicillin and semi-synthetic penicillins. In Brazil, this condition has been observed in 87.7% of CoNS isolates in BSIs,^4^ and thus, vancomycin becomes the choice for the initial treatment for these patients. In CoNS species, an important correlation between oxacillin resistance and decreased susceptibility to glycopeptides has been shown.

*Staphylococcus cohnii* is a microorganism belonging to the CoNS group. It is composed of two major subspecies that are defined on the basis of their characteristics: *Staphylococcus cohnii* subsp. *cohnii* and *Staphylococcus cohnii* subsp. *urealyticus*. It is occasionally a skin colonizer, like *Staphylococcus simulans, Staphylococcus xylosus, Staphylococcus saprophyticus* and *Staphylococcus warneri*; thus, *Staphylococcus cohnii* infection in humans is an unusual event.^5^ They are nonmotile, nonspore-forming, Gram-positive, negative coagulase, aerobic, clustering cocci that produce catalase. Few cases of *Staphylococcus cohnii-*related infection have been described in the literature, It may be responsible for bacteremia relating to catheters, surgical prostheses such as spinal fixation material, acute cholecystitis, brain abscess, endocarditis, pneumonia, urinary tract infection and septic arthritis,^6^ generally presenting with a multiresistant profile in human infections, with nearly 90% of them showing resistance to methicillin.^6^ It is believed to form a genetic resistance reservoir in hospitals. The subspecies differ regarding their colonization environments: *Staphylococcus cohnii* subsp. *cohnii* isolates have more frequently been isolated in hospital than in non-hospital environments, whereas *Staphylococcus cohnii* subsp. *urealyticus* isolates occur more frequently in homes, and are only rarely isolated from hospitalized patients and staff.^6^

Some case series have already reported *Staphylococcus cohnii* samples. An Argentinian hemodialysis center reported 59 strains, of which 18% were oxacillin-resistant, 27% were clindamycin-resistant and none were vancomycin or teicoplanin-resistant; however, no clinical infection charts were reported in relation to this population.^7^ A Turkish study reported an occurrence of staphylococcal true bacteremia (with two positive samples from blood cultures) in general wards, while one case of *Staphylococcus cohnii* bacteremia was reported from an intensive care unit, without reports on infection foci or the antibiotic resistance profile.^8^ A Brazilian survey on blood samples positive for CoNS in a nosocomial setting showed that the prevalence of *Staphylococcus cohnii* was 5.9% (n = 14), with 28.6% susceptibility to oxacillin and 100% susceptibility to vancomycin and teicoplanin.^9^ A Czech case series showed one nosocomial blood culture strain of *Staphylococcus cohnii* subsp. *urealyticus*, with a notable resistance profile, such that it was only sensitive to three of the thirteen antibiotics tested.^6^ An outbreak of infection caused by linezolid-resistant *Staphylococcus cohnii* subsp. *urealyticus* in a Greek intensive care unit was reported, with septicemia in four patients; this was due to selection pressure through linezolid use in case of vancomycin-resistant *Enterococcus faecium*.^10^

## CONCLUSIONS

The reported case is, to our knowledge, the first case of true bacteremia due to *Staphylococcus cohnii* subsp. *urealyticus* caused by an infected pressure ulcer. It shows that this species may be underdiagnosed and should be considered in the differential diagnosis for community-acquired skin infections.
